# Force Sensor Characterization Under Sinusoidal Excitations

**DOI:** 10.3390/s141018454

**Published:** 2014-10-06

**Authors:** Nieves Medina, Jesús de Vicente

**Affiliations:** 1 Centro Español de Metrología, CEM, Calle Alfar 2, 28760 Tres Cantos, Madrid, Spain; 2 Escuela Técnica Superior de Ingenieros Industriales de la Universidad Politécnica de Madrid, ETSII-UPM, Calle de José Gutiérrez Abascal, 2, 28006 Madrid, Spain; E-Mail: jvo@etsii.upm.es

**Keywords:** dynamic force, dynamic calibration, force standard, sinusoidal excitation, rocking motion

## Abstract

The aim in the current work is the development of a method to characterize force sensors under sinusoidal excitations using a primary standard as the source of traceability. During this work the influence factors have been studied and a method to minimise their contributions, as well as the corrections to be performed under dynamic conditions have been established. These results will allow the realization of an adequate characterization of force sensors under sinusoidal excitations, which will be essential for its further proper use under dynamic conditions. The traceability of the sensor characterization is based in the direct definition of force as mass multiplied by acceleration. To do so, the sensor is loaded with different calibrated loads and is maintained under different sinusoidal accelerations by means of a vibration shaker system that is able to generate accelerations up to 100 m/s^2^ with frequencies from 5 Hz up to 2400 Hz. The acceleration is measured by means of a laser vibrometer with traceability to the units of time and length. A multiple channel data acquisition system is also required to simultaneously acquire the electrical output signals of the involved instrument in real time.

## 1. Introduction

Static calibration of force sensors has a well-established procedure, which is fully described in ISO 376 [1], however, many applications of force transducers are in dynamic conditions. Examples are impact testing for security in vehicles, modal testing for structures and materials to check their seismic behavior or robotized systems used in many applications. As it will be described through this article, important effects and corrections have to be taken into consideration for the proper use of a force sensor under dynamic conditions; as a consequence, it is clear that an adequate dynamic characterization of the force sensor is essential.

In order to address these needs, some National Metrology Institutes are currently working in the development of traceable methods to characterise force sensors under dynamic conditions, which are being realized by means of shock and sinusoidal forces. The most recent works for shock forces have been performed by Physicalish-Technische Bundesanstalt (PTB) [2,3] and National Institute of Metrology (NIM) [4]. PTB [5–7] is also very actively working in the field of sinusoidal forces, where National Institute of Standards and Technology (NIST) [8] and Centro Español de Metrología (CEM) [9] have recently started their studies.

The realization of sinusoidal forces is based on the direct definition of force as mass multiplied by acceleration. The force sensor is loaded with different calibrated masses and different accelerations are generated by a vibration shaker system. The acceleration is measured by a laser vibrometer traceable to the unit of length (laser wavelength).

Being a fully dynamic measurement it requires a multichannel data acquisition system in real time. It will acquire electrical output signals from the laser vibrometer, sensor under calibration, and other auxiliary accelerometers. The required signal treatment and calculations will be performed by special software, which has been implemented for this purpose.

The sensor is characterised by its dynamic sensitivity *S*(*f*), which is the ratio of its electrical output signal of the force sensor and the acting dynamic force and it is a function of the frequency *f* of the sinusoidal force. The sensor response may depend on frequency. The sensitivity is going to be a complex quantity (sensor output signal may not be in phase with the applied force), thus, a modulus |S(*f*)| and a phase φ(*f*) will be considered:

(1) $$S(f)=\left|S(f)\right|\cdot {\text{e}}^{j\varphi (f)}$$

When calibrating a force sensor it is also expected a dependency of the sensitivity on the force. This effect is usually observed in calibrations under static conditions. In calibrations under dynamic conditions the sensitivity uncertainty will be larger than the sensitivity uncertainty under static conditions. This difference is such that any possible dependency on the force can be considered negligible if compared with the standard uncertainty, which can be obtained in the determination of the dynamic sensitivity. As a consequence, this work will focus on the dependency of the sensitivity on the excitation frequency and the possible dependency on the force will be considered negligible.

## 2. Description of the System

**Figure 1.**
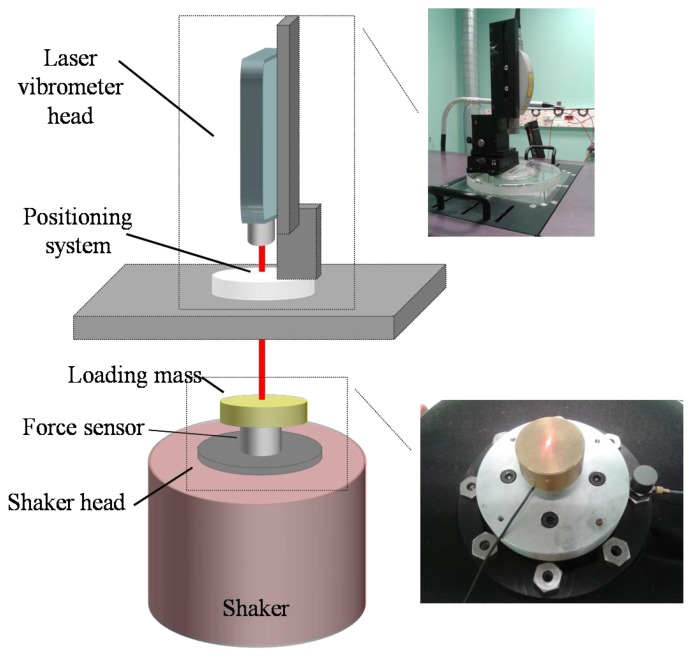
Calibration setup consisting of the shaker with the force sensor mounted on the shaker head and a laser vibrometer for the acceleration measurement with the positioning system for the laser beam orientation. The force sensor is loaded with an additional mass.

In this section the special features of the measurement system implemented at CEM (see Figure 1) for the characterisation of force sensors under sinusoidal excitations are described.

The laser vibrometer (Polytec CLV 2534) measures the velocity on one single spot. It is a modified Mach-Zehnder interferometer and it incorporates a Bragg cell (opto-acoustic modulator). The signal from the photodetector is digitalized and mixed with two phase-shifted π/2 signals with the same frequency used for the opto-acoustic modulation to obtain two baseband signals in separate branches. When they pass through their corresponding low pass filters, two signals are obtained in quadrature. The arctangent calculation and a subsequent numerical differentiation generate the data which contain the information of velocity against time. After passing through a low pass filter and a digital analog converter, a voltage output signal proportional to the velocity of the sinusoidal movement under measurement is obtained from the laser vibrometer. The laser vibrometer traceability is described in [10].

The laser vibrometer includes a system for the adequate positioning of the laser beam on the mass surface according to spherical coordinates, which is located on a special table for placing the laser vibrometer over the shaker (type LDS 726 with power amplifier type PA 2000).

The required loading masses for generating the forces on the force sensor have been manufactured and calibrated to determine their mass and their corresponding uncertainty. The loading masses have nominal values of 0.35, 1, 2, 7.2 and 12.3 kg. The three smaller loading masses are cylinders that can be screwed directly to the sensor under calibration. The two larger ones are cylinders with a cylindrical hole in the center, which are connected to the sensor under calibration by means of a special pressure coupling that can be screwed directly to the sensor under calibration. Depending on the sensor to be calibrated, additional adaptors may be required in order to attach the masses to the sensor or the sensor to the shaker.

The data acquisition system is a NI PXI 1033 module with a 4462 card (24 bits, 204.8 kS/s), which is programmed in Labview. The implemented software samples each signal separately with a rate of 40,000 samples per second. The excitation frequency is directly detected by the software, so the conversion of the output signal of the laser vibrometer as velocity into acceleration is straightforward. In order to minimize the possible noise effect, the sine approximation method is implemented with Labview functions in real time. According to this method every signal *a*(*t_i_*) is fitted according to Equation (2):

(2) $$a(t_{i})=A\cos(2\pi ft_{i})-B\sin(2\pi ft_{i})+C$$

where *i* = 1…*N* and *N* is the total number of measurement samples considered for the approximation. This generates an overdetermined system of equations in which the coefficients *A*, *B* and *C* can be estimated. Amplitude *α̂* and phase *a*_φ_ can then be determined according to Equation (3):

(3) $$\begin{array}{l} \hat{a}=\sqrt{A^{2}+B^{2}}\\ a_{\varphi }=\arctan\left(\frac{-B}{A}\right) \end{array}$$

The implemented software is able to perform Fast Fourier Transforms for each signal in real time as well as determine their total harmonic distortion. When there is no resonant behavior the study of the signals spectra shows that their harmonic components are negligible for orders higher than the third one. In addition, the maximum measured total harmonic distortion has been 2% for frequencies far from resonances.

In order to minimize the effect of harmonics, this method has been implemented, also considering harmonics up to their third order according to Equation (4):

(4) $$\begin{array}{c} a(t_{i})=C+A_{0}\cos(2\pi ft_{i})-B_{0}\sin(2\pi ft_{i})+A_{1}\cos(4\pi ft_{i})-B_{1}(4\pi ft_{i})+\\ A_{2}\cos(6\pi ft_{i})-B_{2}\sin(6\pi ft_{i})+A_{3}\cos(8\pi ft_{i})-B_{3}\sin(8\pi ft_{i}) \end{array}$$

Obviously only the fundamental frequency components are considered for sensitivity determination. This least squares approximation is performed by means of the singular value decomposition method.

## 3. Influence Factors and Corrections

The influences in dynamic calibration of force sensors are different from their static calibration. Effects, such as creep, zero drift and hysteresis, will not appear in dynamic calibration. In this section the influences and corrections for dynamic calibration are described. The study of the influences will be followed by recommendations for a proper measurement and the corrections will be explained to get the correct result for the dynamic sensitivity.

### 3.1. Influence of the Resonance Behavior

As any system under sinusoidal excitation, the system shaker-sensor-loading mass behaves as a mechanical resonator; this is a loading mass *m* connected to the sensor by a spring of stiffness *k*. In order to study this behavior, the accelerations at the top of the mass and the top surface of the shaker have to be measured. This is performed by the laser vibrometer at the top of the mass and an accelerometer (B'K 8305) at the top surface of the shaker. The acquisition system is also used to perform these measurements.

**Figure 2.**
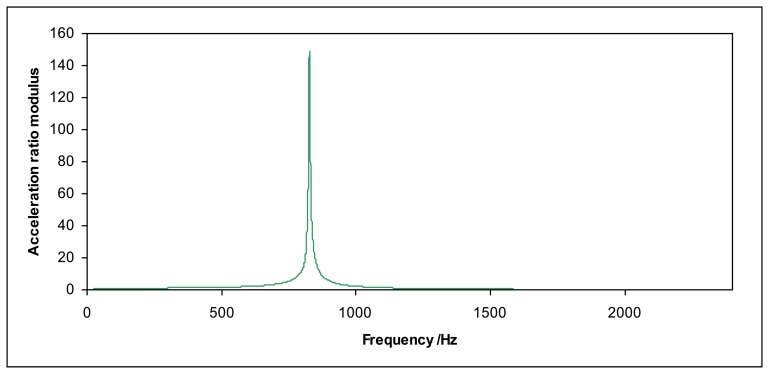
Acceleration ratio modulus for the Kistler 9175B sensor (12.3 kg loading mass).

**Figure 3.**
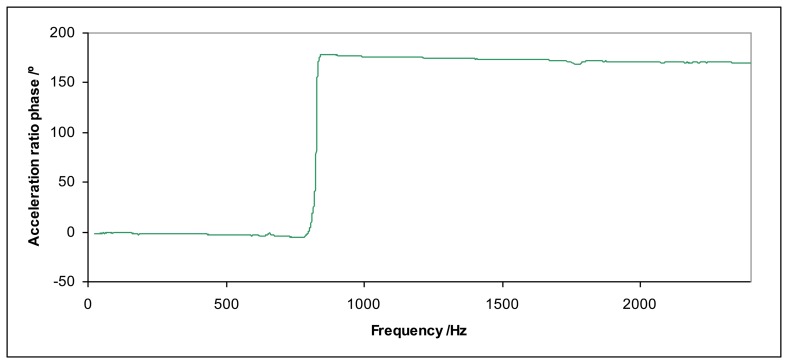
Acceleration ratio phase for the Kistler 9175B sensor (12.3 kg loading mass).

The ratio between the acceleration at the top of the mass and the acceleration at the top surface of the shaker allows determining the resonant frequency *f*_0_ (examples in Figures 2 and 3) for each loading mass *m*. The stiffness *k* for each force sensor can be determined according to Equation (5):

(5) $$k=m{\left(2\pi f_{0}\right)}^{2}$$

This effect is very important because the system becomes unstable close to the resonance frequency, the total harmonic distortion increases and measurements are not reliable. If the excitation acceleration of the shaker is high, the acceleration of the mass can increase so much that the sensor may be seriously damaged. Therefore, measurements close to the resonant frequency *f*_0_ should be avoided.

### 3.2. Influence of Laser Beam Location

**Figure 4.**
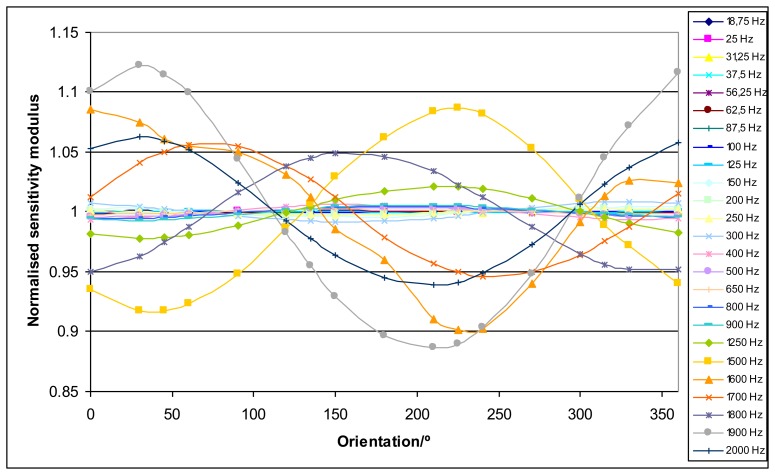
Each curve represents the sensitivity modulus divided by the mean sensitivity modulus for a different excitation frequency *versus* the different orientations of the laser vibrometer beam location around a circumference of radius 1 cm for the Kistler 9175B sensor (2 kg loading mass).

The acceleration of the mass measured by the laser vibrometer depends on the position of its laser beam on the top of the mass. Thanks to the positioning system for the laser vibrometer beam this effect has been studied. The acceleration has been measured over circumferences with different radius that were concentric with the loading mass center, which is coincident with the vertical z axis of the positioning system. The measurements were performed for the orientations 0°, 30°, 45°, 60°, 90°, 120°, 135°, 150°, 180°, 210°, 225°, 240°, 270°, 300°, 315°, 330° and 360°. This last orientation was measured as a repeatability test for the process. The orientation was ensured with an accuracy of 1°. An example of this influence is shown in Figure 4.

**Figure 5.**
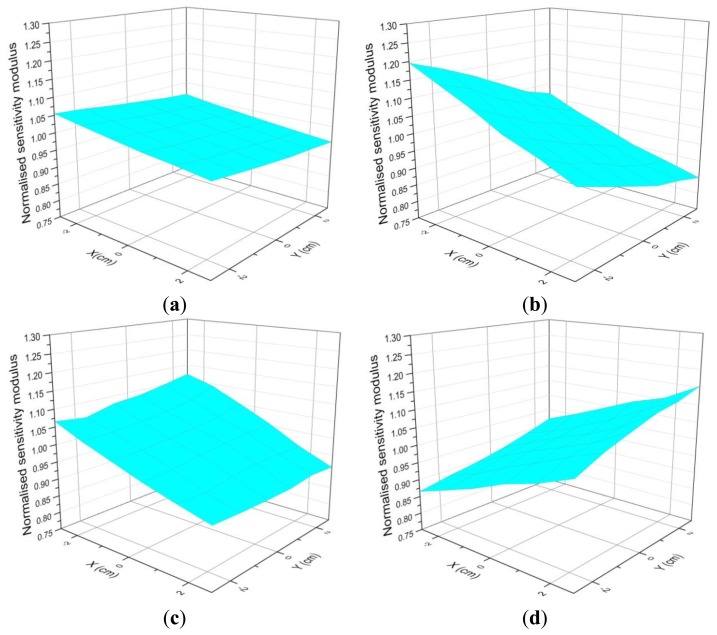
(**a**) 3D plot obtained from interpolation results when the laser beam is at different locations for 1250 Hz; (**b**) 3D plot obtained from interpolation results when the laser beam is at different locations for 1500 Hz; (**c**) 3D plot obtained from interpolation results when the laser beam is at different locations for 1800 Hz; and (**d**) 3D plot obtained from interpolation results when the laser beam is at different locations for 2000 Hz.

As a general result, it has been obtained that the sensitivity as a function of the orientation of the laser vibrometer beam location shows a sinusoidal behavior. Figure 4 shows an example of this effect for the sensitivity modulus, but similar results are also obtained for the phase. This effect is more important as loading mass and frequency increase. The interpolation of the results from these different circumferences plus a radial measurement to connect them has allowed obtaining 3D plots. Some examples of these 3D plots are shown in Figure 5.

The general result for this experiment shows that the system presents rocking motion [11,12]. In order to minimize this rocking motion effect, it is clear that the sensitivity measurement has to be performed with the laser beam being focused on the center of the top of the mass when possible. According to Figure 4 this effect has a sinusoidal behavior; therefore this centered value will be the correct value. This center is difficult to be determined exactly so, in practice, measurements are performed in very small circumferences as close as possible to this center. In fact the mean value of two radial measurements 180° apart provide a good estimate of the correct value, but performing several measurements around a circumference provides a better estimate with smaller uncertainty according to a method described in Section 4.3.

### 3.3. Influence of the Shaker Movement Effects

**Figure 6.**
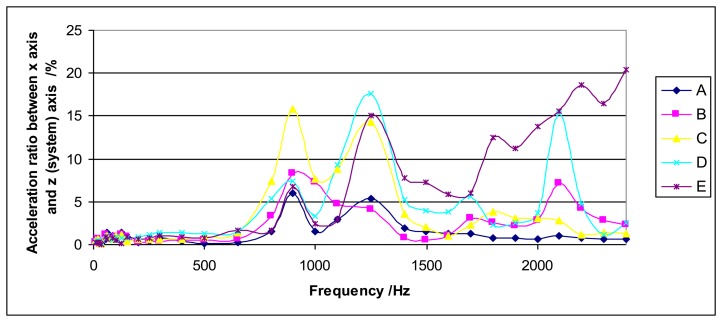
Ratio between the acceleration measured in the x axis and the acceleration measured in the *z* axis (system axis) *versus* excitation frequency for different spots on the measurement system: Upper part of the loading mass (A), lower part of the loading mass (B), mass-sensor coupling (C), sensor surface (D) and shaker upper surface (E). The sensor type is Interface 1610 and the loading mass is 2 kg.

**Figure 7.**
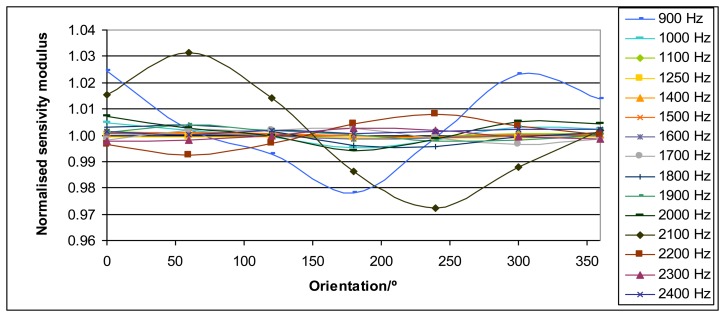
Each curve represents the modulus of sensitivity divided by the mean value for a different excitation frequency *versus* the different orientations of the laser vibrometer beam measuring location (minimum radius) for the Interface 1610 sensor (2 kg loading mass).

As the shaker is the source of the required vibration, it is clear that any disturbance in its movement will affect it. Ideally the shaker should only move in the vertical direction but, in practice, it also generates transversal acceleration. This transversal acceleration has been measured by the laser vibrometer accelerometer in two transversal directions (*x* and *y*) for different heights of the system: Upper part of the loading mass, lower part of the loading mass, mass-sensor coupling, sensor surface and shaker upper surface. An example is shown in Figure 6. It shows some interesting peaks in the acceleration ratio corresponding to frequencies 900 Hz, 1250 Hz and 2100 Hz. There are similar results for *y* axis. The peak at 1250 Hz may be explained for its closeness to the resonant frequency of the sensor (1193 Hz). The peaks at 900 Hz and 2100 Hz are related to the rocking motion effect, as it can be deduced from Figure 7.

In conclusion, there is a high correlation between transverse acceleration and the increase of rocking motion; therefore, when the shaker generates transverse acceleration at a certain frequency, the increase of rocking motion will notably affect the measurement.

On the other hand, the acceleration in the *x* axis increases with frequency from 1600 Hz, but just for the shaker upper surface. This is not a problematic issue as for sensitivity determination the acceleration is measured at the top surface of the loading mass.

### 3.4. Influence of Mounting

In order to minimize rocking motion effects and other disturbances a proper mounting has to be ensured, otherwise unexpected peaks in the sensitivity curve may appear for certain frequencies. Special care has to be taken to ensure that the contact between pieces is made by means of flat surfaces. The torque that is applied to connect the different parts has also to be sufficient, if not specified by the manufacturer, and carefully controlled.

On the other hand, a triboelectric effect caused by the cable movement in relation to the sensor may appear. In order to minimize this effect, the sensor cable should be tied down to a non-movable part of the system. It has to be stretched but not tense in such away that no tension appears to the connector. If these recommendations are followed this effect is negligible.

### 3.5. Influence of the Magnetic Field Generated by the Shaker

The movement of the shaker head is generated by the Lorentz force. This force is generated by the sinusoidal current with intensity *I*(ω) that passes through a coil attached to the shaker head. The shaker head is affected by a static magnetic field with intensity *B*′, which is generated by an external coil. The sinusoidal current with intensity *I*(ω) also generates a sinusoidal magnetic field with intensity *B*(ω) at the same time. The effect of this magnetic field generates a current density *J*′ in any conductor with permeability μ and conductivity σ according to the Maxwell Equation (6):

(6) $$\begin{array}{c} \nabla \times \text{J}\prime =-j\omega \sigma \text{B}\prime \\ \nabla \times \text{B}\prime =\mu \text{J}\prime \end{array}$$

where the time dependency according to a sinusoidal variation with angular frequency ω (ω = 2π*f*) has been taken into account. As a consequence of the solution for this system of equations, an induced current *I′*(ω) is generated inside the conductor according to Equation (7):

(7) $$I\prime \approx \frac{1}{j\omega \sigma \mu }$$

This current may be induced in the sensor and it is important for low excitation frequencies. Its effects are more noticeable the lower the load on the sensor is, as this induced current does not depend on the load.

**Figure 8.**
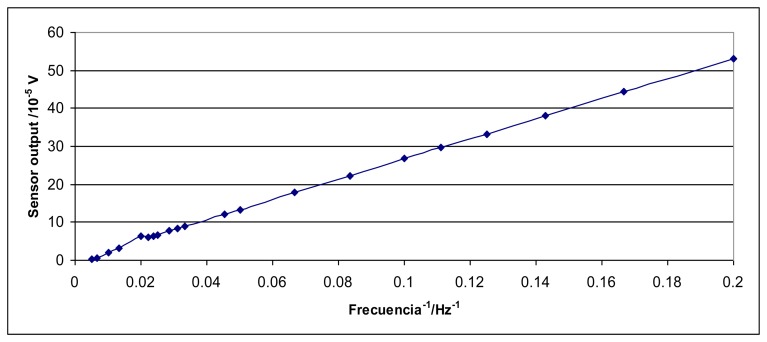
Output for the Interface 1610 sensor, when it was hanging over a magnet attached to the shaker head, *versus* the inverse of the shaker excitation frequency.

As an experimental confirmation of this effect, a strong magnet was attached to the shaker head and the sensor was put hanging over this magnet without contact. When the shaker head was moving, the sensor output signal was inversely proportional to the inverse of the excitation frequency and the signal phase was 90° in relation to the shaker excitation signal. Figure 8 shows an example of this behavior.

**Figure 9.**
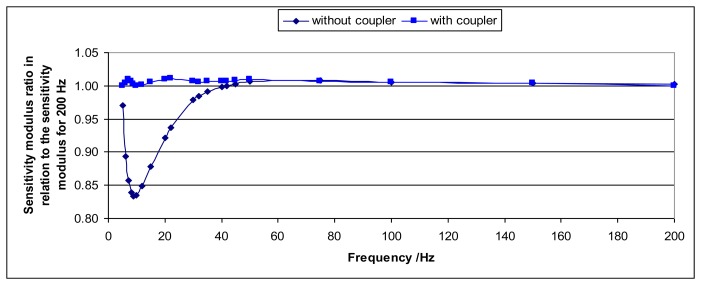
The figure represents the ratio between the sensitivity modulus and the sensitivity modulus for 200 Hz *versus* the excitation frequency for the Interface 1610 sensor. One curve is for the case without the coupler and the other curve is the case when the coupler is used.

**Figure 10.**
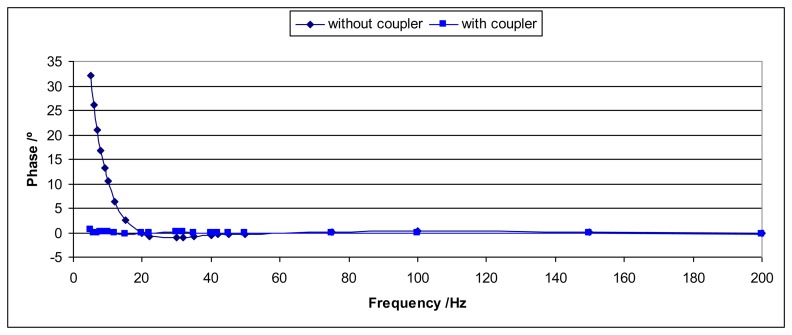
The figure represents the phase *versus* the excitation frequency for the Interface 1610 sensor. One curve is for the case without the coupler and the other case is for the case when the coupler is used.

One way to avoid this effect is to increase the distance between the sensor and the shaker head by means of a coupler. In this way, the influence of the induced magnetic field is negligible. For the shaker that has been used during these experiments it has been enough to increase the distance to 17 cm with a mechanical coupler. Figures 9 and 10 show the comparison of the sensitivity modulus and the phase for two cases: when the coupler is used and when it is not used. The sensitivity modulus for 200 Hz is used as a reference because the effect is negligible for this frequency (and higher frequencies). It is clear that, thanks to the coupler, the magnetic effects are negligible.

### 3.6. Sensor Internal Mass Correction

The sensor has an internal mass that contributes to the sensor load. As a consequence, the sensor sensitivity has to be corrected according to Equation (8):

(8) $$S_{m}=S\cdot \frac{m}{m+m_{\mathrm{int}}}$$

where *S_m_* is the correct sensitivity, *S* is the sensitivity without correction, *m* is the loading mass and *m*_int_ is the sensor internal mass. The importance of this correction is inversely proportional to the loading mass weight. The sensor internal mass is determined considering the sensitivity constant for different loads in the range of excitation frequency where other possible corrections are negligible; this is the medium frequency range.

In static calibration this sensor internal mass has no effect because the sensor output signal is adjusted to be zero for the case when no force is applied.

### 3.7. Pressure Coupling Correction

**Figure 11.**
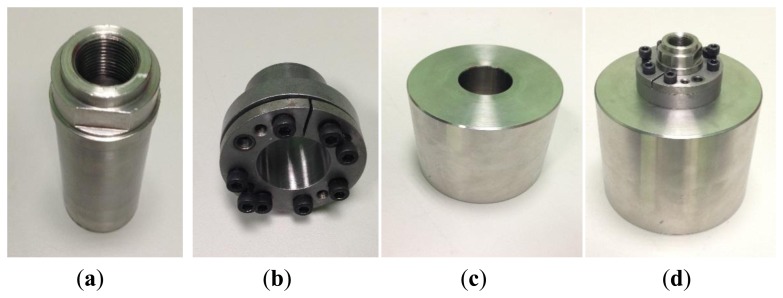
The figure represents the two parts of the pressure coupling element, (**a**) and (**b**), one large cylindrical loading mass, (**c**), and the whole assembly, (**d**).

For the larger masses (7.2 kg and 12.3 kg) a pressure-coupling element is used (see Figure 11 for details). This element connects the loading mass with the sensor not by means of a screw but with pressure.

This kind of coupling can be very imperfect and causes the loading mass motion to be different from that of the coupling element and sensor, which are moving with the same acceleration because they are connected by means of a screw. As a consequence, the force sensitivity determined with the acceleration measured on the coupling element, *S*_1_, and the force sensitivity determined with the acceleration measured on the loading mass, *S*_2_, are different. The correct sensitivity in this case *S_m_* has to be determined according to Equation (9), where *m*_1_ is the mass of the coupling element, *m*_2_ is the mass of the loading mass and *m*_int_ is the sensor internal mass:

(9) $$S_{m}=\frac{m_{1}+m_{2}}{\frac{m+m_{\mathrm{int}}}{S_{1}}+\frac{m_{2}}{S_{2}}}$$

In order to determine the correct sensitivity it has to be taken into consideration the fact that mass and coupling element move independently when subjected to the same excitation. This correct sensitivity is directly defined according to Equation (10):

(10) $$S_{m}=\frac{V}{(m_{1}+m_{\mathrm{int}})a_{1}+m_{2}a_{2}}$$

where *V* is the sensor output signal and *a*_1_ and *a*_2_ are respectively the loading mass acceleration and the coupling element acceleration. Since the sensor output signal *V* is affected by the total load *m*_1_ + *m*_2_ + *m*_int_ and the data acquisition system takes into account the total load too but for the sensor internal mass *m*_int_, two sensitivities are determined by the software according to Equation (11):

(11) $$\begin{array}{l} S_{1}=\frac{V}{(m_{1}+m_{2})a_{1}}\\ S_{2}=\frac{V}{(m_{1}+m_{2})a_{2}} \end{array}$$

During the measurement process each one of these sensitivities is determined separately, so that the correct sensitivity *S_m_* can be easily determined according to Equation (9) as a function of *S*_1_, *S*_2_, *m*_1_, *m*_int_ and *m*_2_. This equation is derived directly from Equations (10) and (11).

### 3.8. Mass Rigidity Correction

For this correction, loading masses are considered to behave as rigid bars according to the theory described in [13], in which the longitudinal waves are transmitted through the system with velocity *c*:

(12) $$c=\sqrt{\frac{Y}{\rho }}$$

where *Y* is the Young modulus and ρ is the density for the mass material. Assuming that the spatial deformation variation is zero on the top surface of the loading mass, and this deformation is originated by the sinusoidal excitation force with angular frequency ω, the corrected sensitivity *S_c_* can be obtained from the previous sensitivity *S_m_* according to Equation (13):

(13) $$S_{c}=S_{m}\cdot \left(\frac{\omega (L+L_{0})\sqrt{\frac{\rho }{Y}}}{\sin\left(\omega (L+L_{0})\sqrt{\frac{\rho }{Y}}\right)}\right)$$

where *L* is the loading mass length and *L*_0_ comes from the transmission of the longitudinal waves through the sensor. *L*_0_ depends on the sensor and it is typically zero, but it may be different from zero especially for sensors with big dimensions.

Mass rigidity correction is more important for high frequencies and long loading masses.

## 4. Uncertainty Determination

The uncertainty determination is performed according to JCGM 100 [14]. The type B contributions come from the uncertainty in use of the different parts of the system: laser vibrometer, loading masses and coupling elements, data acquisition system and the influence of the temperature in the force sensor. The type B uncertainty contributions are listed separately for sensitivity modulus (see Table 1) and sensitivity phase (see Table 2) in the tables below.

When the pressure coupling is used (7.2 kg and 12.3 kg) and Equation (9) has to be used for corrections, no additional uncertainty contribution is required. This result follows from applying JCGM 100 for Equation (9) and assuming maximum correlation for mass and sensitivity uncertainty contributions for both the loading mass and the pressure coupling as they are mutually dependent.

The type A contributions include the contribution for the sine approximation, the repeatability of measurements, the laser beam location and the system reproducibility. Each contribution will be explained below in more detail.

### 4.1. Sine Approximation Uncertainty Contribution

The sine approximation method has been implemented in Labview by means of the singular value decomposition method. This method has been implemented in such a way that the mean squared error *s*^2^ for the approximation can be obtained directly from the software for each measurement.

Each output signal *a* obtained either from the laser vibrometer or a the force sensor has been fitted to a sinusoidal curve with fundamental frequency *f* that takes into consideration harmonics up to the third order. As the maximum total harmonic distortion is 2% it will be enough considering only the first harmonic for uncertainty determination according to Equation (14):

(14) $$S(t_{i})≅A\cos(2\pi ft_{i})-B\sin(2\pi ft_{i})+C$$

Applying the least squares approximating philosophy for *n* samples, the coefficients *A*, *B* and *C* are obtained as described in Equation (15):

(15) $$\begin{array}{l} A=\frac{2}{n}\underset{i}{\text{∑}}S(t_{i})\cos(2\pi ft_{i})\\ B=-\frac{2}{n}\underset{i}{\text{∑}}S(t_{i})\sin(2\pi ft_{i})\\ C=\frac{1}{n}\underset{i}{\text{∑}}S(t_{i}) \end{array}$$

These results have been obtained under the following assumptions. Firstly, sums can be approximated to integrals because the number of samples is very high (40,000 per measurement) and secondly, only complete periods can be considered as the cycles number is very high (10 is the minimum value as the minimum exciting frequency is 10 Hz and the largest error would be half a cycle in 10 cycles, this would be a 5% error). For the uncertainty determination of the coefficients, the results (see Equation (16)) are obtained with the same previous assumptions:

(16) $$\begin{array}{l} u^{2}(A)=s^{2}\left(\frac{2}{n}\right)\\ u^{2}(B)=s^{2}\left(\frac{2}{n}\right)\\ u^{2}(C)=\left(\frac{s^{2}}{n}\right) \end{array}$$

As a consequence, the uncertainty contributions for the sensitivity modulus *α̂* and the sensitivity phase *a*_φ_ would be determined according to Equation (17):

(17) $$\begin{array}{l} u^{2}(\hat{a})=u^{2}\left(\sqrt{A^{2}+B^{2}}\right)=s^{2}\left(\frac{2}{n}\right)\\ u^{2}\left(a_{\varphi }\right)=u^{2}\left(a\tan\left(\frac{-B}{A}\right)\right)=s^{2}\left(\frac{2}{n}\right)\left(\frac{1}{A^{2}+B^{2}}\right) \end{array}$$

Sensitivity is proportional to the ratio between the laser vibrometer (referred with *lv* subscript) and the force sensor (referred with *fs* subscript) output signals. As the signals are acquired by the same data acquisition system, maximum correlation is assumed and the relative uncertainty contribution for the sensitivity modulus *w*(|*S*|) and the uncertainty contribution for the sensitivity phase *u*(φ) in degrees (°) can be determined according to Equation (18):

(18) $$\begin{array}{c} w{\left(\left|S\right|\right)}_{\text{aprox}}=\sqrt{\left(\frac{s_{lv}^{2}}{A_{lv}^{2}+B_{lv}^{2}}+\frac{s_{fs}^{2}}{A_{fs}^{2}+B_{fs}^{2}}\right)\left(\frac{2}{n}\right)}\\ u{\left(\left|\varphi \right|\right)}_{\text{aprox}}=\frac{180}{\pi }\sqrt{\left(\frac{s_{lv}^{2}}{A_{lv}^{2}+B_{lv}^{2}}+\frac{s_{fs}^{2}}{A_{fs}^{2}+B_{fs}^{2}}\right)\left(\frac{2}{n}\right)} \end{array}$$

### 4.2. Repeatibility

Ten measurements are performed in the same measurement conditions (laser beam position, loading mass and excitation frequency). The standard deviation of these measurements is considered as the repeatability contribution and it is determined directly by the implemented Labview software. Tests have been performed with fifty measurements instead of ten obtaining similar results, so ten measurements are considered to be sufficient.

### 4.3. Laser Beam Location

As described in Section 3.2 the acceleration of the mass measured by the laser vibrometer depends on the position of its laser beam on the top of the mass. As a general result, it has been obtained that the sensitivity (modulus and phase) as a function of the orientation of the laser vibrometer beam location shows a sinusoidal behavior. In order to minimize this effect, it is clear that the sensitivity measurement has to be performed with the laser beam being focused as close as possible to the center of the top of the mass. This center is difficult to be determined exactly, thus, in practice, measurements are performed in very small circumferences as close as possible to this center. As this effect shows a sinusoidal behavior a sensitivity value (modulus or phase) that corresponds to this center will be the correct value. Consequently the straightforward method to obtain the correct sensitivity modulus and the sensitivity phase is the approximation of these values to sinusoidal functions *y* of the orientation θ according to Equation (19):

(19) y=m+Ksin(θ+α)

where *K*, *m* and α are parameters to be determined. The only useful one will be *m*, the centered value, which will be determined by a weighted least squares approximation; this is, Equation (20) has to be a minimum:

(20) $$\sum =\sum_{i}\frac{{\left(m+K\sin(\theta_{i}+\phi )-y_{i}\right)}^{2}}{\sigma_{i}^{2}}$$

where σ*_i_* are obtained by the combination of the uncertainty contributions for repeatability and the sine approximation. Following the usual procedure for least squares approximations it is obtained that the centered value *m* is provided by Equation (21):

(21) $$m=\frac{\underset{i}{\text{∑}}\frac{\left(y_{i}-K\sin(\theta_{i}+\alpha )\right)}{\sigma_{i}^{2}}}{\underset{i}{\text{∑}}\frac{1}{\sigma_{i}^{2}}}$$

and its uncertainty is provided by Equation (22):

(22) $$u^{2}(m)=\frac{1}{\underset{i}{\text{∑}}\frac{1}{\sigma_{i}^{2}}}$$

As the parameters *K* and α are unknown the centered value *m* is obtained by a minimization recursive procedure with the maximum variation in sensitivity for the initial value for *K* and zero as the initial value for α. In practice the value for *m* will not be very different from its weighted mean, as *K* is very small, the expression is evaluated in 2π and σ*_i_* are similar among them.

### 4.4. System Reproducibility

**Figure 12.**
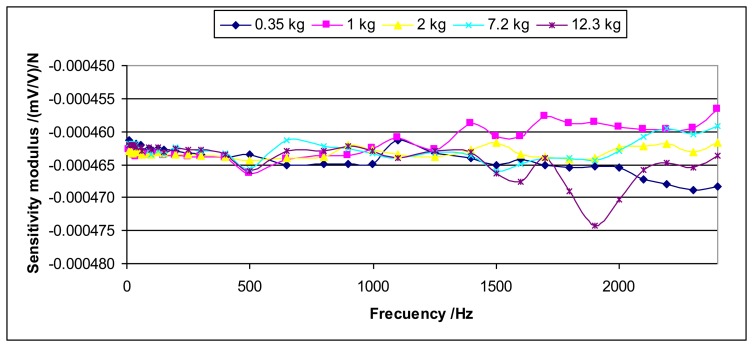
Each curve represents the sensitivity modulus for each load *versus* frequency for the Interface 1610 sensor.

**Figure 13.**
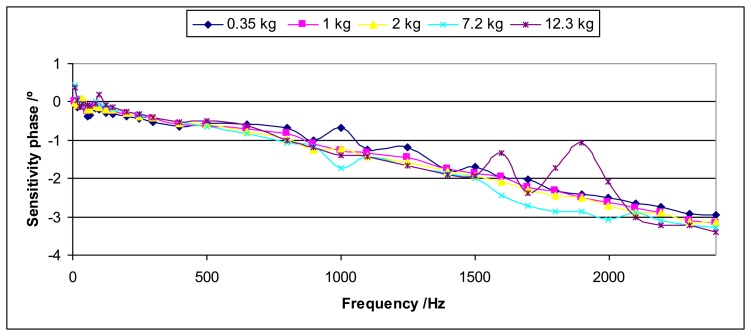
Each curve represents the sensitivity phase for each load *versus* frequency for the Interface 1610 sensor.

For dynamic measurements the sensitivity will be independent from the load. This uncertainty contribution considers the lack of reproducibility for the different loads. This may be caused by the mounting and the shaker effects among other effects. Figures 12 and 13 show an example of this lack of reproducibility for the sensitivity modulus and the sensitivity phase, respectively.

This uncertainty contribution will be determined as the standard deviation of the mean for each excitation frequency among the values obtained for the different loads. This determination will be applied to the sensitivity modulus as well as the sensitivity phase.

The values obtained for this uncertainty contribution can be very important especially for high frequencies.

## 5. Sensor Characterization

As a general rule it has been found that the sensitivity modulus in the frequency range under study does not depend on the excitation frequency but, on the contrary, there is a clear linear dependency of the sensitivity phase with the excitation frequency. Their uncertainty depends on excitation frequency, so a weighted mean can be used to determine sensitivity modulus and a weighted linear approximation can be used to determine the sensitivity phase.

**Figure 14.**
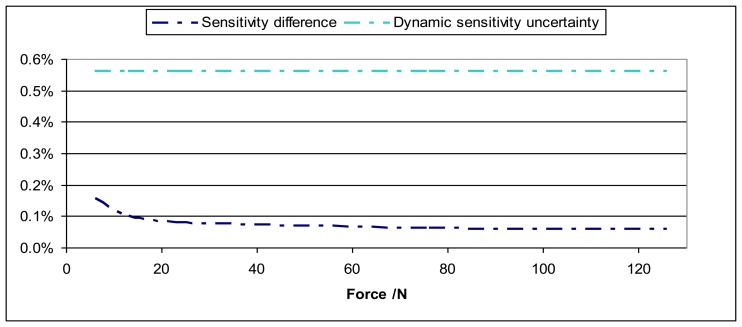
One curve represents the sensitivity difference between static and dynamic calibration and the other curve represents the dynamic sensitivity uncertainty *versus* applied force for the Interface 1610 sensor.

Calibration results for sensitivity modulus have been compared for dynamic and static conditions. The static calibration has been performed according to ISO 376 using a deadweight primary force machine as standard [15] with relative expanded uncertainty 2 × 10^−5^. These results have been found to be fully in agreement as the possible difference between static and dynamic sensitivities is less than the dynamic sensitivity expanded uncertainty. Figure 14 shows an example of these results.

Dynamic calibration allows determining a set of parameters, which are essential for the characterization of the sensor and its further proper use. These parameters are the sensor internal mass, which is a very important correction for low forces and the stiffness *k*, which is used to determine the resonant frequency as a function of the force, where the sensor may presents problems.

## 6. Conclusions

This work has established a procedure to characterize force sensors under sinusoidal excitations using a primary standard as the source of traceability.

It has been studied the influence factors that are typical for dynamic sinusoidal measurements, such as the rocking motion, the transversal acceleration and the magnetic field from the shaker and the mounting effects. Recommendations have been explained to minimise their effects. Specifically the sensor cable should be attached to a non movable part of the system and it has to be stretched but not tense, special care has to be taken to ensure that the contact between pieces is made by means of flat surfaces, and the torque which is applied to connect the different parts also has to be carefully controlled and sufficient, if not specified by the manufacturer. In order to avoid the magnetic effects caused by the movement of the shaker head, it may be necessary to increase the distance between the sensor and the shaker head by means of a special coupler.

The internal mass sensor, the pressure coupling, if used, and the mass rigidity cause corrections that have to be performed in order to get accurate results. These corrections have been described in detail in this work. In particular, the use of the pressure coupling has demonstrated to be inconvenient, as double measurement work is required; although it does not suppose an increase to the uncertainty. On the other hand, it is better to have short than long cylinders as loading masses to minimize the mass rigidity correction.

Although there is no clear dependency of the sensitivity modulus with the excitation frequency, and this value is the same as the one obtained under static conditions; its dynamic characterization is essential. It is important because it provides the corrections for the sensor internal mass, the stiffness *k* to determine the resonance frequency for the different forces and the adequate torque, if not supplied by the manufacturer. On the other hand, the dynamic calibration also provides the sensitivity phase as a function of the excitation frequency and the calibration uncertainty for sensitivity modulus and phase for each excitation frequency, which will be very useful as the lower limit for the uncertainty during the current use of the sensor under dynamic conditions.

## Figures and Tables

**Table 1.** Type B uncertainty contributions for the sensitivity modulus.

**Quantity**	**Uncertainty Contribution**
Laser vibrometer	0.14%
Mass	0.02%
Data acquisition system	0.05%
Temperature influence in the sensor	Up to 0.06%

**Table 2.** Type B uncertainty contributions for the sensitivity phase.

**Quantity**	**Uncertainty Contribution**
Laser vibrometer	0.23°
Data acquisition system	1.5 × 10^−5^ /Hz
